# Regaining function via peripheral nerve surgery

**DOI:** 10.3171/2022.10.FOCVID22108

**Published:** 2023-01-01

**Authors:** Mariano Socolovsky, Zarina S. Ali, Brandon W. Smith, Kevin N. Swong

**Affiliations:** 1 Neurosurgery, University of Buenos Aires, Buenos Aires, Argentina;; 2 Department of Neurosurgery, University of Pennsylvania, Philadelphia, Pennsylvania;; 3 Department of Neurosurgery, Duke University Medical Center, Durham, North Carolina; and; 4 Department of Neurological Surgery, Northwestern University Feinberg School of Medicine, Chicago, Illinois

When we were finishing the final review of this issue of *Neurosurgical Focus: Video* dedicated to peripheral nerve surgery, we received the absolutely unexpected and terribly sad notice of the passing of our team leader, Lynda J. S. Yang (1967–2022) (Fig. 1).

**FIG. 1:**
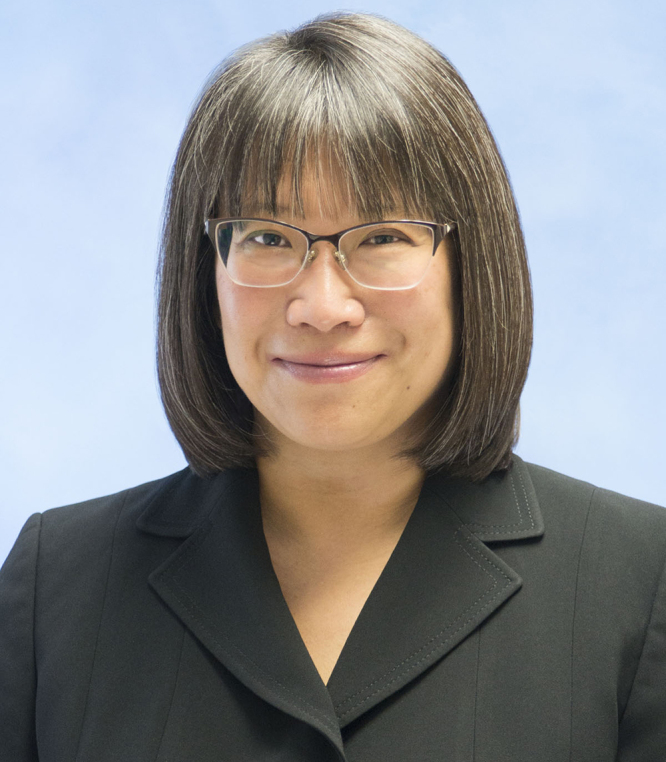
Photograph of Dr. Lynda J. S. Yang. © University of Michigan Department of Neurosurgery, published with permission.

Lynda formed this working group mirroring her leadership principles: she was an expert in peripheral nerve surgery and valued the importance of imparting this knowledge to her mentees by establishing an environment that afforded her mentees the opportunity to work with experienced leaders in the field, as she indeed was. Throughout her career, Lynda combined a very high level of scientific excellence and rigor with a tremendous generosity when teaching and sharing her knowledge. This was mirrored in the jovial spirit embodied in who she was as a person. She wisely knew how to blend education and training with a permeating positivity and a vast generosity, leading her to be a highly esteemed friend and colleague for those who had the privilege to be involved in both academic and social spheres with her.

Peripheral nerve surgery is unique in neurosurgery, as it allows for restoration of lost neurological function by way of regenerating and repairing damaged axons. We hope the viewership of this issue of *Neurosurgical Focus: Video* will find these principles reflected in the innovative surgical techniques described by the contributors, with the hope of providing neurosurgical patients with benefits not previously thought possible.

This work follows the spirit that Lynda embodied in creativity, generosity, and scientific rigor, which grievously now transforms into one of her last scientific contributions of a very productive and fruitful academic life. We hope the viewership will surely enjoy it and reference it for practical approaches to restoring and repairing lost neurologic function, while keeping the spirit of our team leader, Lynda J. S. Yang, in their thoughts.

## Acknowledgments

We acknowledge the contributions of Kate Wan-Chu Chang, MA, MS, Senior Researcher, Brachial Plexus and Peripheral Nerve Program, Michigan Medicine.

## Disclosures

The authors report no conflict of interest.

